# Book Review: Safe Is Not Enough: Better Schools for LGBTQ Students (Youth Development and Education Series)

**DOI:** 10.3389/fpsyg.2021.704995

**Published:** 2021-06-23

**Authors:** Alex Siu Wing Chan

**Affiliations:** Department of Applied Social Sciences, The Hong Kong Polytechnic University, Hong Kong, China

**Keywords:** school inclusion, social inclusion and exclusion, school policies, LGBT young people, education system and inequality

Two suggested state policies in the United States will include specific security for students who identify as lesbian, gay, bisexual, transgender, queer, and questioning (LGBTQ). These new regulations have been introduced in response to several state and school system efforts to identify and enforce laws or initiatives aimed at ensuring the protection of LGBTQ students in educational institutions. Over the last 10 years, studies have revealed that LGBTQ teenagers are a disadvantaged demographic, and that their traumatic school encounters often add to that insecurity. This public policy paper discusses the study that has been conducted on certain federal, regional, and national regulations and practices. The author reviews the studies on sexuality formation, with an emphasis on the rising proportion of adolescents who “come out” or reveal their LGBTQ sexuality to everyone else throughout their time at school. Educational institutions are mostly threatening settings for LGBTQ students; such data is weighed against studies on the effects of impaired academic performance, cognitive, and mental well-being (Chan et al., 2021). We then examine interventions in teaching and learning that have been linked to LGBTQ (as well as all) students' welfare (Chan, 2021b). Safe Is Not Enough demonstrates that school systems should provide extensive resources for the healthy growth of LGBTQ students in order to foster more supportive classroom cultures. Michael Sadowski in this book discusses current approaches like developing an LGBTQ-friendly syllabus, promoting a welcoming environment throughout the entire school for LGBTQ students, having grown-ups who could serve as counselors and authority figures, as well as implementing appropriate household and neighborhood engagement campaigns, incorporating case studies from classes, campuses, and regions around the nation.

For more than two decades, researchers have reported that LGBT adolescents face increased levels of abuse, rejection, and violence in educational institutions than their straight counterparts. Such adverse events have been published in the United States and a number of different European nations (Palkki and Caldwell, 2018; Scannapieco et al., 2018). These analyses recognized four distinct types of abuse: anti-gay words, oral ridicule, mental oppression, and actual violent behavior, and established that most LGBTQ students face name-calling, abusive behavior, intimidation, and violence at school. For instance, in the GLSEN's National School Climate Survey, Kosciw et al. (2012) discovered that almost all LGBT students encountered offensive comments at school, with more than 75% hearing them regularly or intensively. Additionally, the writers discovered that more than 80% of LGBT students revealed experiencing oral harassment, more than 40% revealed experiencing bodily harassment and more than 20% revealed experiencing physical assault as a result of their gender identity.

Though improvement on LGBTQ problems in educational institutions has been sluggish, schools in certain areas of the nation have taken steps to create healthier and more accepting environments for LGBTQ students. Usually, colleges and universities do so by updating their anti-bullying guidelines and creating gay-straight student alliances (GSAs). However, transforming campuses into environments in which LGBTQ students could reach their full capacity requires not just a reactive strategy. Sadowski discusses ways in which teaching staff could render their classrooms more conducive to LGBTQ students' healthy growth as well as educational attainment in Safe Is Not Enough.

This book begins by introducing audiences to three prominent LGBTQ programs in educational institutions that concentrate on child protection: supportive anti-bullying programs, Safe Zones (which is demonstrated by Safe Zone markings), and Gay-Straight Alliances, known as GSAs. Although every one of them is critical for students, Sadowski suggests that teachers strive for even greater inclusion through activities like integrated syllabuses, effective psychological well-being strategies, and enhanced career advancement. The audience is subsequently guided by a slew of examples highlighting specific services, educational institutions, and regions in which LGBTQ integration extends past basic security concerns. All contexts are vividly depicted with the use of an incredibly simple narration format. The audience is further directed to an index that contains the complete list of resources employed in a variety of examples. On the whole, the book concisely illustrates instances of educational institutions moving past sheer security for LGBTQ students, while also introducing services which can be utilized by a broad range of academic staff, such as teachers, supervisors, student groups, and campus psychological support specialists (such as curriculums for a Common Core-compliant LGBTQ integrated course, and an overview of an LGBTQ academic therapy community). Context details are presented in a concise manner. Sadowski begins by defining the term LGBTQ (lesbian, gay, bisexual, transgender, queer, and questioning), followed by a short section explaining why this word was chosen. He further debunks many myths of bisexual and transgender rejection in LGBTQ literary works.

NPR commonly employs the term LGBT to apply to individuals that identify as “lesbian, gay, bisexual, or transgender.” Sadowski prefixes “queer” with a “Q.” This study does the same thing. Sadowski's book contains several accounts. He is a specialist in teenage personality formation with a particular emphasis on students who identify as LGBTQ (lesbian, gay, bisexual, transgender, and queer). Sadowski interviewed teaching staff across the United States to discover how they are incorporating LGBTQ-friendly strategies into their institutions' syllabuses and environments. Sadowski also attacks tactics which include protection as the ultimate target. Safety is a crucial aim, and he describes it as “a critical baseline from which all subsequent work must follow.”

“Safe is not enough: Better schools for LGBTQ students” should be studied by both teachers and education administrators and should be used in college learning and social service practice. Indeed, considering the increasing prominence of LGBTQ individuals as involved (Chan, 2021a), valued, and visible fellow citizens, this book is especially important since it addresses the causes and consequences of LGBTQ discriminatory practices (Chan, 2021c). I definitely appreciate it because it promotes community cohesion and inclusion in the academic environment.

## Author Contributions

The author confirms being the sole contributor of this work and has approved it for publication.

## Conflict of Interest

The author declares that the research was conducted in the absence of any commercial or financial relationships that could be construed as a potential conflict of interest.
